# Pyridinium-2-carboxyl­ate–benzene-1,2-diol (1/1)

**DOI:** 10.1107/S1600536809043207

**Published:** 2009-10-23

**Authors:** Cuong Quoc Ton, Michael Bolte

**Affiliations:** a Institut für Organische Chemie der Goethe-Universität Frankfurt, Max-von-Laue-Strasse 7, D-60438 Frankfurt am Main, Germany; b Institut für Anorganische Chemie der Goethe-Universität Frankfurt, Max-von-Laue-Strasse 7, D-60438 Frankfurt am Main, Germany

## Abstract

The title compound, C_6_H_5_NO_2_·C_6_H_6_O_2_, crystallizes with one pyridinium-2-carboxyl­ate zwitterion and one mol­ecule of benzene-1,2-diol in the asymmetric unit. The crystal structure is characterized by alternating mol­ecules forming zigzag chains running along the *a* axis: the mol­ecules are connected by O—H⋯O and N—H⋯(O,O) hydrogen bonds.

## Related literature

For co-crystallization experiments, see: Ton & Bolte (2005 ▶); Tutughamiarso *et al.* (2009 ▶).
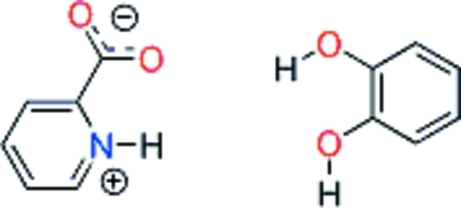


## Experimental

### 

#### Crystal data

C_6_H_5_NO_2_·C_6_H_6_O_2_
*M* *_r_* = 233.22
Orthorhombic, 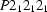

*a* = 6.9710 (14) Å
*b* = 6.9855 (14) Å
*c* = 21.806 (4) Å
*V* = 1061.9 (4) Å^3^
*Z* = 4
Mo *K*α radiation
μ = 0.11 mm^−1^
*T* = 173 K
0.21 × 0.18 × 0.16 mm

#### Data collection

Stoe IPDSII two-circle diffractometer
Absorption correction: none
11928 measured reflections
1196 independent reflections
1105 reflections with *I* > 2σ(*I*)
*R*_int_ = 0.081

#### Refinement

*R*[*F* ^2^ > 2σ(*F* ^2^)] = 0.096
*wR*(*F* ^2^) = 0.197
*S* = 1.23
1196 reflections
155 parameters
H-atom parameters constrained
Δρ_max_ = 0.44 e Å^−3^
Δρ_min_ = −0.34 e Å^−3^

### 

Data collection: *X-AREA* (Stoe & Cie, 2001 ▶); cell refinement: *X-AREA*; data reduction: *X-AREA*; program(s) used to solve structure: *SHELXS97* (Sheldrick, 2008 ▶); program(s) used to refine structure: *SHELXL97* (Sheldrick, 2008 ▶); molecular graphics: *XP* in *SHELXTL-Plus* (Sheldrick, 2008 ▶); software used to prepare material for publication: *PLATON* (Spek, 2009 ▶) and *SHELXL97*.

## Supplementary Material

Crystal structure: contains datablocks I, global. DOI: 10.1107/S1600536809043207/ng2670sup1.cif

Structure factors: contains datablocks I. DOI: 10.1107/S1600536809043207/ng2670Isup2.hkl

Additional supplementary materials:  crystallographic information; 3D view; checkCIF report

## Figures and Tables

**Table 1:** Hydrogen-bond geometry (Å, °)

*D*—H⋯*A*	*D*—H	H⋯*A*	*D*⋯*A*	*D*—H⋯*A*
O1—H1⋯O11^i^	0.84	1.84	2.655 (6)	163
O2—H2⋯O12	0.84	1.89	2.662 (7)	153
N1—H31⋯O12	0.91	2.16	2.617 (7)	110
N1—H31⋯O1	0.91	2.18	2.984 (7)	147

Symmetry code: (i) 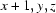.

**Fig. 1.**
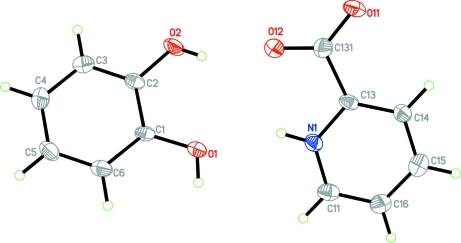
A view of the molecular structure of the title compound, with the atom-numbering scheme. Displacement ellipsoids are drawn at the 50% probability level and H atoms are shown as small spheres of arbitrary radii.

**Fig. 2.**
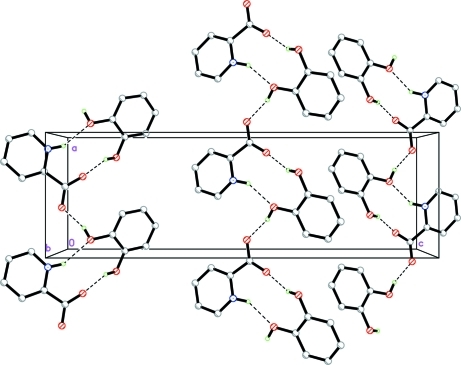
Part of the crystal packing of the title compound. Hydrogen bonds are shown as dashed lines.

## supplementary crystallographic information

### Comment

The aim of our research is the cocrystallization of two small organic compounds in order to examine the hydrogen bonds formed between hydrogen-bond acceptors and hydrogen-bond donors (Ton & Bolte, 2005; Tutughamiarso *et al.*, 2009). When pyridinecarboxaldehyde and 1,2-dihydroxybenzene were mixed in order to obtain a hydrogen bonded supermolecular complex, it turned out that the aldehyd had been oxidized to the carboxylic acid. The title compound crystallizes with one pyridinium-2-carboxylate zwitterion and one molecule of benzene-1,2-diol in the asymmetric unit. The crystal structure is characterized by alternating molecules forming zigzag chains running along the *a* axis. The molecules are connected by O—H···N and O—H···O hydrogen bonds.

### Experimental

40 mg pyridinecarboxaldehyde and 40 mg 1,2-dihydroxybenzene were diluted in 2 ml diethyl ether in a nitrogen atmosphere. After five weeks a brown precipitate emerged from the mixture. On the surface white crystals has been sedimented, one of which was used for structure determination. It turned out that the pyridinecarboxaldehyde had been oxidized to the carboxylic acid.

### Refinement

Hydrogen atoms were located in a difference Fourier map but those bonded to C and O were included in calculated positions [C—H = 0.93 - 0.99 Å] and refined as riding [*U*_iso_(H) = 1.2*U*_eq_(C) or *U*_iso_(H) = 1.5*U*_eq_(*O*,*C*_methyl_)]. H atoms bonded to N were freely refined. Due to the absence of anomalous scatterers, the absolute structure could not be determined and 808 Friedel pairs were merged.

### Crystal data

**Table:**

C_6_H_5_NO_2_·C_6_H_6_O_2_	*F*(000) = 488
*M**_r_* = 233.22	*D*_x_ = 1.459 Mg m^−^^3^
Orthorhombic, *P*2_1_2_1_2_1_	Mo *K*α radiation, λ = 0.71073 Å
Hall symbol: P 2ac 2ab	Cell parameters from 6345 reflections
*a* = 6.9710 (14) Å	θ = 3.5–24.3°
*b* = 6.9855 (14) Å	µ = 0.11 mm^−^^1^
*c* = 21.806 (4) Å	*T* = 173 K
*V* = 1061.9 (4) Å^3^	Block, colourless
*Z* = 4	0.21 × 0.18 × 0.16 mm

### Data collection

**Table:**

Stoe IPDSII two-circle diffractometer	1105 reflections with *I* > 2σ(*I*)
Radiation source: fine-focus sealed tube	*R*_int_ = 0.081
graphite	θ_max_ = 25.8°, θ_min_ = 3.1°
ω scans	*h* = −8→8
11928 measured reflections	*k* = −8→8
1196 independent reflections	*l* = −26→25

### Refinement

**Table:**

Refinement on *F*^2^	Secondary atom site location: difference Fourier map
Least-squares matrix: full	Hydrogen site location: inferred from neighbouring sites
*R*[*F*^2^ > 2σ(*F*^2^)] = 0.096	H-atom parameters constrained
*wR*(*F*^2^) = 0.197	*w* = 1/[σ^2^(*F*_o_^2^) + (0.0513*P*)^2^ + 3.5668*P*] where *P* = (*F*_o_^2^ + 2*F*_c_^2^)/3
*S* = 1.23	(Δ/σ)_max_ = 0.001
1196 reflections	Δρ_max_ = 0.44 e Å^−^^3^
155 parameters	Δρ_min_ = −0.34 e Å^−^^3^
0 restraints	Extinction correction: *SHELXL97* (Sheldrick, 2008), Fc^*^=kFc[1+0.001xFc^2^λ^3^/sin(2θ)]^-1/4^
Primary atom site location: structure-invariant direct methods	Extinction coefficient: 0.036 (6)

### Special details

**Table:**

Geometry. All e.s.d.'s (except the e.s.d. in the dihedral angle between two l.s. planes) are estimated using the full covariance matrix. The cell e.s.d.'s are taken into account individually in the estimation of e.s.d.'s in distances, angles and torsion angles; correlations between e.s.d.'s in cell parameters are only used when they are defined by crystal symmetry. An approximate (isotropic) treatment of cell e.s.d.'s is used for estimating e.s.d.'s involving l.s. planes.
Refinement. Refinement of *F*^2^ against ALL reflections. The weighted *R*-factor *wR* and goodness of fit *S* are based on *F*^2^, conventional *R*-factors *R* are based on *F*, with *F* set to zero for negative *F*^2^. The threshold expression of *F*^2^ > σ(*F*^2^) is used only for calculating *R*-factors(gt) *etc*. and is not relevant to the choice of reflections for refinement. *R*-factors based on *F*^2^ are statistically about twice as large as those based on *F*, and *R*- factors based on ALL data will be even larger.

### Fractional atomic coordinates and isotropic or equivalent isotropic displacement parameters (Å^2^)

**Table:**

	*x*	*y*	*z*	*U*_iso_*/*U*_eq_	
O1	1.1064 (7)	0.3802 (7)	0.0845 (2)	0.0257 (11)	
H1	1.2093	0.4153	0.0679	0.039*	
O2	0.8174 (6)	0.1866 (8)	0.1466 (2)	0.0295 (12)	
H2	0.7994	0.2756	0.1214	0.044*	
C1	1.1455 (9)	0.3029 (9)	0.1410 (3)	0.0200 (13)	
C2	0.9985 (9)	0.2048 (10)	0.1716 (3)	0.0220 (13)	
C3	1.0330 (10)	0.1145 (11)	0.2274 (3)	0.0259 (14)	
H3	0.9332	0.0460	0.2473	0.031*	
C4	1.2160 (10)	0.1249 (11)	0.2542 (3)	0.0303 (16)	
H4	1.2399	0.0649	0.2925	0.036*	
C5	1.3612 (9)	0.2231 (10)	0.2244 (3)	0.0280 (15)	
H5	1.4850	0.2302	0.2424	0.034*	
C6	1.3273 (9)	0.3110 (10)	0.1686 (3)	0.0254 (14)	
H6	1.4284	0.3777	0.1487	0.031*	
O11	0.3875 (7)	0.5038 (7)	0.0125 (2)	0.0307 (12)	
O12	0.6497 (8)	0.4131 (10)	0.0643 (3)	0.0516 (18)	
N1	0.8827 (8)	0.5092 (8)	−0.0246 (2)	0.0222 (12)	
H31	0.9086	0.4912	0.0160	0.027*	
C11	1.0169 (10)	0.5516 (10)	−0.0666 (3)	0.0260 (15)	
H11	1.1479	0.5226	−0.0591	0.031*	
C13	0.6922 (9)	0.5494 (9)	−0.0326 (3)	0.0208 (13)	
C14	0.6360 (10)	0.6404 (9)	−0.0853 (3)	0.0237 (14)	
H14	0.5049	0.6725	−0.0914	0.028*	
C15	0.7722 (10)	0.6856 (10)	−0.1299 (3)	0.0275 (15)	
H15	0.7347	0.7491	−0.1665	0.033*	
C16	0.9629 (10)	0.6368 (11)	−0.1202 (3)	0.0301 (17)	
H16	1.0559	0.6628	−0.1510	0.036*	
C131	0.5649 (10)	0.4815 (11)	0.0200 (3)	0.0283 (15)	

### Atomic displacement parameters (Å^2^)

**Table:**

	*U*^11^	*U*^22^	*U*^33^	*U*^12^	*U*^13^	*U*^23^
O1	0.015 (2)	0.032 (2)	0.030 (2)	−0.002 (2)	0.0002 (18)	0.006 (2)
O2	0.015 (2)	0.033 (3)	0.041 (3)	−0.005 (2)	−0.003 (2)	0.007 (2)
C1	0.017 (3)	0.015 (3)	0.028 (3)	−0.007 (3)	0.002 (3)	−0.001 (3)
C2	0.017 (3)	0.020 (3)	0.029 (3)	−0.004 (3)	0.000 (3)	−0.003 (3)
C3	0.025 (3)	0.026 (3)	0.027 (3)	−0.002 (3)	0.006 (3)	−0.002 (3)
C4	0.032 (4)	0.034 (4)	0.026 (3)	0.001 (3)	−0.004 (3)	0.006 (3)
C5	0.019 (3)	0.035 (4)	0.030 (3)	0.000 (3)	−0.004 (3)	−0.003 (3)
C6	0.019 (3)	0.029 (3)	0.028 (3)	−0.005 (3)	0.003 (3)	−0.003 (3)
O11	0.016 (2)	0.043 (3)	0.033 (2)	−0.002 (2)	0.002 (2)	0.002 (3)
O12	0.024 (3)	0.086 (5)	0.044 (3)	0.015 (3)	0.007 (2)	0.033 (3)
N1	0.019 (3)	0.025 (3)	0.023 (2)	0.004 (3)	0.001 (2)	0.000 (2)
C11	0.022 (3)	0.022 (3)	0.034 (3)	0.000 (3)	0.005 (3)	−0.002 (3)
C13	0.015 (3)	0.015 (3)	0.033 (3)	−0.001 (2)	0.002 (3)	0.000 (3)
C14	0.019 (3)	0.025 (3)	0.027 (3)	0.000 (3)	−0.002 (3)	0.002 (3)
C15	0.038 (4)	0.021 (3)	0.024 (3)	−0.001 (3)	−0.002 (3)	−0.002 (3)
C16	0.028 (4)	0.034 (4)	0.028 (3)	−0.001 (3)	0.004 (3)	0.002 (3)
C131	0.028 (4)	0.028 (3)	0.029 (3)	0.004 (3)	0.003 (3)	0.005 (3)

### Geometric parameters (Å, °)

**Table:**

O1—C1	1.374 (8)	O11—C131	1.257 (8)
O1—H1	0.8397	O12—C131	1.229 (9)
O2—C2	1.381 (7)	N1—C11	1.342 (9)
O2—H2	0.8392	N1—C13	1.368 (8)
C1—C2	1.401 (9)	N1—H31	0.9123
C1—C6	1.404 (9)	C11—C16	1.365 (10)
C2—C3	1.391 (9)	C11—H11	0.9500
C3—C4	1.405 (9)	C13—C14	1.370 (9)
C3—H3	0.9500	C13—C131	1.526 (9)
C4—C5	1.384 (10)	C14—C15	1.396 (10)
C4—H4	0.9500	C14—H14	0.9500
C5—C6	1.383 (9)	C15—C16	1.389 (10)
C5—H5	0.9500	C15—H15	0.9500
C6—H6	0.9500	C16—H16	0.9500
			
C1—O1—H1	109.4	C11—N1—H31	123.7
C2—O2—H2	109.1	C13—N1—H31	110.1
O1—C1—C2	118.3 (5)	N1—C11—C16	119.3 (7)
O1—C1—C6	123.2 (5)	N1—C11—H11	120.4
C2—C1—C6	118.5 (6)	C16—C11—H11	120.4
O2—C2—C3	117.5 (6)	N1—C13—C14	118.6 (6)
O2—C2—C1	121.7 (6)	N1—C13—C131	113.9 (6)
C3—C2—C1	120.7 (6)	C14—C13—C131	127.4 (6)
C2—C3—C4	119.8 (6)	C13—C14—C15	119.7 (6)
C2—C3—H3	120.1	C13—C14—H14	120.2
C4—C3—H3	120.1	C15—C14—H14	120.2
C5—C4—C3	119.6 (6)	C16—C15—C14	119.4 (6)
C5—C4—H4	120.2	C16—C15—H15	120.3
C3—C4—H4	120.2	C14—C15—H15	120.3
C6—C5—C4	120.5 (6)	C11—C16—C15	120.0 (7)
C6—C5—H5	119.8	C11—C16—H16	120.0
C4—C5—H5	119.8	C15—C16—H16	120.0
C5—C6—C1	120.9 (6)	O12—C131—O11	128.6 (7)
C5—C6—H6	119.6	O12—C131—C13	115.6 (6)
C1—C6—H6	119.6	O11—C131—C13	115.8 (6)
C11—N1—C13	123.0 (6)		
			
O1—C1—C2—O2	−0.7 (10)	C11—N1—C13—C14	−1.3 (10)
C6—C1—C2—O2	−178.3 (6)	C11—N1—C13—C131	177.8 (6)
O1—C1—C2—C3	176.4 (6)	N1—C13—C14—C15	1.5 (9)
C6—C1—C2—C3	−1.2 (10)	C131—C13—C14—C15	−177.4 (6)
O2—C2—C3—C4	178.6 (6)	C13—C14—C15—C16	0.2 (10)
C1—C2—C3—C4	1.4 (10)	N1—C11—C16—C15	2.5 (11)
C2—C3—C4—C5	−0.8 (11)	C14—C15—C16—C11	−2.3 (11)
C3—C4—C5—C6	0.1 (11)	N1—C13—C131—O12	5.1 (9)
C4—C5—C6—C1	0.1 (11)	C14—C13—C131—O12	−176.0 (7)
O1—C1—C6—C5	−177.0 (6)	N1—C13—C131—O11	−175.2 (6)
C2—C1—C6—C5	0.5 (10)	C14—C13—C131—O11	3.7 (10)
C13—N1—C11—C16	−0.8 (10)		

### Hydrogen-bond geometry (Å, °)

**Table:**

*D*—H···*A*	*D*—H	H···*A*	*D*···*A*	*D*—H···*A*
O1—H1···O11^i^	0.84	1.84	2.655 (6)	163
O2—H2···O12	0.84	1.89	2.662 (7)	153
N1—H31···O12	0.91	2.16	2.617 (7)	110
N1—H31···O1	0.91	2.18	2.984 (7)	147

Symmetry codes: (i) *x*+1, *y*, *z*.
